# Study on the inhibitory effect of geniposide on coxsackievirus and its mechanism

**DOI:** 10.3389/fphar.2026.1785779

**Published:** 2026-04-02

**Authors:** Jianbo Xia, Zuliang Shi, Mengxin Shen

**Affiliations:** Department of Laboratory Medicine, Maternal and Child Health Hospital of Hubei Province, Tongji Medical College, Huazhong University of Science and Technology, Wuhan, China

**Keywords:** antiviral activity, coxsackievirus B3, geniposide, molecular docking, network pharmacology

## Abstract

**Background:**

Coxsackievirus infection can cause various diseases such as myocarditis and encephalitis, posing a serious threat to human health. However, there are currently no specific therapeutic drugs or effective vaccines for Coxsackievirus infection, so exploring new anti-Coxsackievirus agents is of great significance. Geniposide, an iridoid glycoside component from traditional Chinese medicine Gardenia jasminoides, has shown multiple pharmacological activities, but its antiviral effect against Coxsackievirus and related mechanisms remain unclear.

**Method:**

This study verified the antiviral activity of geniposide against coxsackievirus *in vitro*, and combined network pharmacology with molecular docking to explore the candidate targets and pathways underlying its effects against coxsackievirus B3 (CVB3).

**Results:**

*In vitro* experiments confirmed that geniposide exhibited significant inhibitory activity against CVB3. Compared with the virus control group, geniposide treatment significantly reduced the degree of pathological changes in HEp-2 and Vero cells infected with CVB3 and effectively inhibited virus plaque formation in a concentration-dependent manner. The 50% effective concentrations (EC_50_) of geniposide in HEp-2 and Vero cells were 165.2 μg/mL and 142.5 μg/mL, respectively. Network pharmacology analysis identified AKT1, STAT1, MMP9, CASP3, MAPK14, and MAPK1 as core potential targets of geniposide against CVB3. Molecular docking results predicted that geniposide could form stable binding conformations with these six core targets, providing a theoretical molecular basis for its antiviral effect.

**Conclusion:**

This study confirmed that geniposide inhibits CVB3 replication *in vitro* in a concentration-dependent manner. Network pharmacology and molecular docking predicted that its anti-CVB3 activity may be associated with binding to core targets including AKT1, STAT1, MMP9, CASP3, MAPK14, and MAPK1. These results provide a hypothetical framework for further mechanistic validation of geniposide against CVB3. This study offers experimental evidence supporting geniposide as a potential anti-coxsackievirus lead compound for future preclinical research, and also provides a novel direction for the prevention and treatment of coxsackievirus infection.

## 1 Introduction

Coxsackievirus, a non-enveloped picornavirus of the genus Enterovirus, is classified into group A (CVA) and group B (CVB) based on its biological characteristics, with group A viruses mainly causing herpangina and hand-foot-mouth disease, and group B viruses tending to induce myocarditis, encephalitis and other diseases (Zhao et al., 2020; Weng et al., 2025). In particular, group B Coxsackieviruses have a strong tropism for tissues such as the heart and pleura, inducing pleural pain, myocarditis, and pericarditis (Zhang et al., 2023). Among group B viruses, Coxsackievirus B3 (CVB3) is recognized as one of the main pathogens of viral myocarditis and dilated cardiomyopathy (Weng et al., 2025). In recent years, viral myocarditis has emerged as a major public health threat to adolescent health (Zhang et al., 2023). However, there is currently no effective vaccine (Mone et al., 2023), and no specific therapeutic drugs are available clinically (Yan et al., 2024). Thus, developing effective drugs against Coxsackievirus infection holds important clinical value and research significance.

Geniposide is an iridoid glycoside compound and the main active constituent of Gardenia jasminoides, a traditional Chinese medicine. It has been recognized as the quality control marker for the fruit of Gardenia jasminoides in the Chinese Pharmacopoeia (Shan et al., 2017; Gong et al., 2024). In this study, geniposide was selected as the sole research compound based on the following systematic considerations: As a highly abundant bioactive component in Gardenia jasminoides, geniposide possesses stable physicochemical properties, which can ensure the reproducibility of experiments (Li et al., 2019); Pharmacokinetic studies have confirmed that geniposide is the main component absorbed into the blood after oral administration of Gardenia jasminoides (Yang et al., 2012), and it exhibits favorable tissue distribution in target organs such as the heart, which is crucial for targeting CVB3-induced myocarditis (Li et al., 2019); Modern pharmacological studies have confirmed its significant anti-inflammatory (Li et al., 2024) and antioxidant effects (Han et al., 2024). In recent years, the antiviral activity of geniposide has also attracted increasing attention. It has been confirmed to exhibit antiviral activity against a variety of viruses, including influenza virus (Zhang et al., 2024) and white spot syndrome virus (WSSV) (Huang et al., 2022). But no research has reported its activity against Coxsackievirus or its role in improving viral myocardial damage. Based on this, this study hypothesizes that geniposide may have potential anti-Coxsackievirus activity.

Network pharmacology, based on systems biology theory, regulates signaling pathways through multiple approaches, enabling effective prediction of drug targets and pathways involved in disease treatment, and providing important technical support for elucidating drug mechanisms (Yuan et al., 2022). This study combines network pharmacology with *in vitro* experimental validation to screen potential targets and pathways of geniposide against coxsackievirus, providing a new direction for antiviral drug development. Molecular docking, a structure-based computational method, plays a key role in drug discovery. It simulates interactions between small-molecule ligands and target proteins to predict the optimal binding conformation and minimum free energy for forming stable complexes (Uppathi et al., 2025). This technology relies on computer simulation, significantly reducing experimental material consumption and repetitive operations, and has become an important tool in drug discovery and molecular simulation research (Singab et al., 2024).

In this study, we aim to address the critical knowledge gap regarding geniposide’s anti-CVB3 activity. We first evaluate its *in vitro* antiviral efficacy by assessing cytoprotection and inhibition of viral plaque formation in CVB3-infected HEp-2 and Vero cells. Using network pharmacology, we identify potential targets of geniposide involved in CVB3 infection and validate these interactions via molecular docking. This study integrated experimental and computational biology approaches to evaluate the *in vitro* anti-CVB3 activity of geniposide and predict its potential targets. The results provide a scientific basis for geniposide as a candidate lead compound against CVB3-related diseases, and also offer a reference for novel therapeutic strategies against coxsackievirus infection.

## 2 Materials and methods

### 2.1 Preparation of virus, cells, and drugs

The CVB3 strain was provided by the Institute of Virology, Wuhan University, and stored at −80 °C. HEp-2 and Vero cell lines were subcultured in the laboratory of the same institute. The culture system was Dulbecco’s Modified Eagle Medium (DMEM, Gibco, Grand Island, NY, United States) supplemented with 10% fetal bovine serum (Gibco), 100 U/mL penicillin (Gibco), and 100 μg/mL streptomycin (Gibco). Cells were cultured in a 37 °C incubator with 5% CO_2_. Geniposide was purchased from Shanghai Jinsui Biotechnology Co., Ltd. The powder was first dissolved in dimethyl sulfoxide (DMSO, Sigma-Aldrich, St. Louis, MO, United States) to a final concentration of 0.1%, and then diluted with ultrapure water to prepare a 1 mg/mL stock solution, which was stored at 4 °C for future use. All CVB3-related experiments were performed in a Biosafety Level 2 (BSL-2) laboratory, in compliance with relevant national biosafety regulations.

### 2.2 Determination of drug cytotoxicity

The 50% cytotoxic concentration (CC50) of geniposide on HEp-2 and Vero cells was measured by MTT assay. Both cell types were seeded in 96-well plates and treated with geniposide at final concentrations of 100, 200, 400, 600, and 800 μg/mL, followed by incubation at 37 °C with 5% CO_2_. After incubation, detection was performed according to the instructions of the MTT assay kit (Sigma-Aldrich). Absorbance at 570 nm was measured using a Multiskan™ GO microplate reader (Thermo Fisher Scientific, Waltham, MA, United States), and CC50 was calculated.

### 2.3 Observation of inhibitory effect on viral replication

Well-grown Vero and HEp-2 cells were seeded in cell culture plates and cultured until a confluent monolayer was formed. After discarding the culture medium, cells were washed once with PBS. Then the cells were infected with CVB3 at a MOI of 0.01 and after 2 h of adsorption, the virus solution was discarded. The experimental groups were added with maintenance medium containing geniposide at concentrations of 100, 200, and 300 μg/mL, while the virus control group and normal control group were given maintenance medium without drug. The normal control group was not inoculated with virus. All groups were further incubated at 37 °C in a 5% CO_2_ incubator for 48 h. Morphological changes of cells in each group were observed under an inverted microscope (CKX-41, Olympus, Japan), and the inhibitory effect of the drug on virus-induced cytopathic effect (CPE) was evaluated.

### 2.4 Plaque reduction assay

Vero and HEp-2 cells were seeded in cell culture plates and cultured until confluent monolayers formed. After discarding the medium, cells were washed once with PBS. The cells were infected with CVB3(100 PFU/well) and after 2 h of adsorption, the virus solution was discarded. The experimental groups were treated with methyl cellulose overlay medium containing geniposide at different concentrations (100, 200, and 300 μg/mL). The overlay medium consisted of 1% methyl cellulose, DMEM, and 2% fetal bovine serum (FBS). The virus control group and normal control group were given the same overlay medium without geniposide. All groups were cultured in an incubator at 37 °C with 5% CO_2_ for 48 h. After discarding the overlay medium, cells were fixed and stained with 1% crystal violet solution at room temperature. After drying, scanned visualization of CVB3 plaques in HEp-2 and Vero cells was observed using a BenQ SZW3300V scanner (BenQ; Suzhou, Jiangsu, China), then the plaque numbers were counted, and the 50% effective concentration (EC50) of the drug against CVB3 was calculated.

### 2.5 Network pharmacology analysis

The SMILES ID and 2D structure of geniposide were obtained from the PubChem database (https://pubchem.ncbi.nlm.nih.gov/) (Li Q. et al., 2025). Its targets were predicted using the Swiss Target Prediction database (http://www.swisstargetprediction.ch/) and Pharm Mapper database (http://www.lilab-ecust.cn/pharmmapper/), and imported into Cytoscape 3.9.1 software to construct a “drug component-target” network. Using “Coxsackievirus B3″ as the keyword, CVB3-related targets were retrieved from the GeneCards database (https://www.genecards.org/) and OMIM database (https://www.omim.org/). The intersection of drug targets and virus-related targets was obtained using the Venny 2.1 online tool, and a Venn diagram was drawn. Submit the intersecting targets to the STRING database (https://string-db.org/) and construct a protein-protein interaction (PPI) network with a high confidence score cutoff of 0.7. Only the protein interaction pairs with a score of no less than 0.7 were retained, and the low-confidence association data were excluded. The established PPI network was then imported into Cytoscape 3.9.1 for visual analysis, and the top 10 core targets were screened out based on the degree value, a key metric for assessing node importance in the network. The DAVID database (https://david.ncifcrf.gov/) was used for GO functional enrichment analysis (including biological processes [BP], molecular functions [MF], and cellular components [CC]) and KEGG pathway enrichment analysis of the intersection targets. With P < 0.05 as the threshold, the top 10 significantly enriched results were selected, and pathway bubble charts were drawn using the Bioinformatics Online Platform (http://www.bioinformatics.com.cn/).

### 2.6 Molecular docking verification

The 2D structure of geniposide was obtained from the PubChem database, and the 3D structure of the corresponding target protein was retrieved from the PDB database (http://www.rcsb.org/) based on the core target gene names. Target proteins were preprocessed (e.g., water removal, hydrogenation) using AutoDockTools 1.5.6 and PyMOL 2.3 software. Geniposide was imported into ChemDraw 3D 2.0 for energy minimization and saved in PDB format. Molecular docking was performed between geniposide and target proteins to calculate binding energy, evaluate binding activity and stability, and predict the interaction between geniposide and core targets.

### 2.7 Statistical analysis

Statistical analysis was performed using GraphPad Prism version 10.5.0 (GraphPad Software, San Diego, California, United States). To determine statistically significant differences between groups, one-way ANOVA was performed, followed by Dunnett’s multiple comparison tests. A p-value <0.05 was considered statistically significant.

## 3 Results

### 3.1 Cytotoxicity of geniposide

**FIGURE 1:**
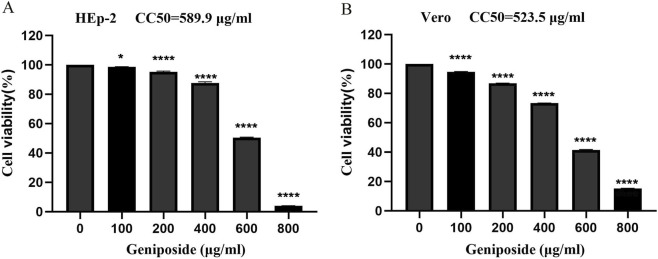
Cytotoxicity of geniposide on HEp-2 and Vero cells. **(A,B)** HEp-2 and Vero cells were exposed to geniposide at different concentrations. Cell viability was assessed via the MTT method, and the CC50 value of geniposide was computed through software analysis. (n = 3; *p < 0.05, **p < 0.01, ***p < 0.001, ****p < 0.0001).

The cytotoxic effect of geniposide on HEp-2 and Vero cells was determined using the MTT assay. As shown in Figures 1A,B, cell viability decreased gradually in a concentration-dependent manner, indicating that the cytotoxicity of geniposide increased with rising concentrations. Based on cell viability corresponding to different drug concentrations, the 50% cytotoxic concentrations (CC_50_) of geniposide were calculated to be 589.9 μg/mL (95% CI: 565.1–611.3 μg/mL) in HEp-2 cells and 523.5 μg/mL (95% CI: 489.0–556.8 μg/mL) in Vero cells, respectively.

### 3.2 Antiviral activity of geniposide

**FIGURE 2:**
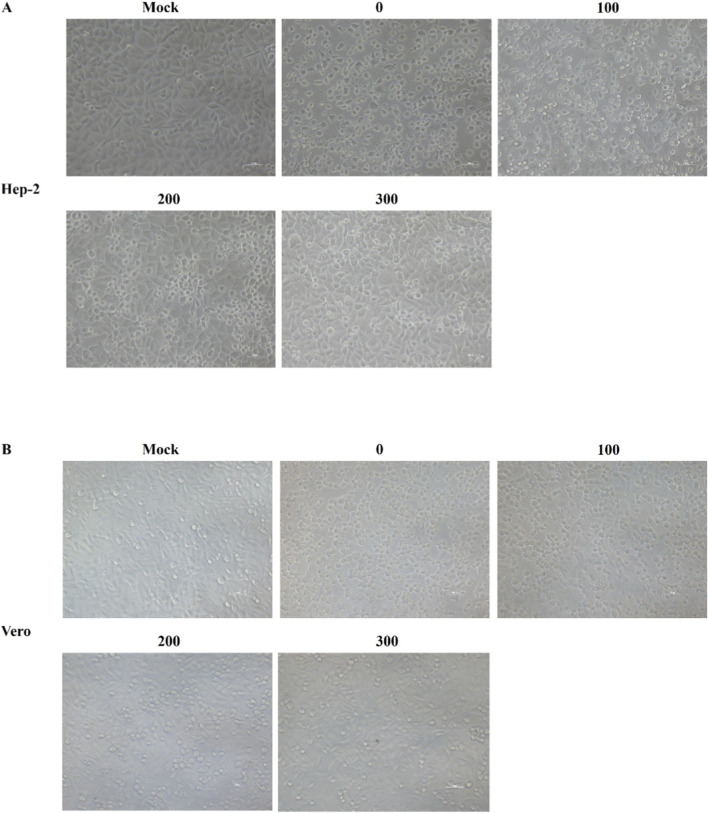
Effects of geniposide on CVB3-infected HEp-2 and Vero cells. **(A)** Microscopic images of HEp-2 cells treated with different concentrations of geniposide after CVB3 infection. **(B)** Microscopic images of Vero cells treated with different concentrations of geniposide after CVB3 infection. Scale bar, 50 μm.

Observation of the protective effect of geniposide on Coxsackievirus-infected cells (Figures 2A,B) showed that HEp-2 and Vero cells in the virus control group exhibited obvious pathological changes, including cell rounding, shrinkage, detachment, and fragmentation. After treatment with geniposide at different concentrations, the degree of virus-induced cytopathic effect (CPE) was significantly attenuated in a concentration-dependent manner and cell morphology was closer to that of the normal control group. These results indicate that geniposide exhibits significant inhibitory activity against Coxsackievirus *in vitro*.

### 3.3 Inhibitory effect of geniposide on viral plaque formation

**FIGURE 3:**
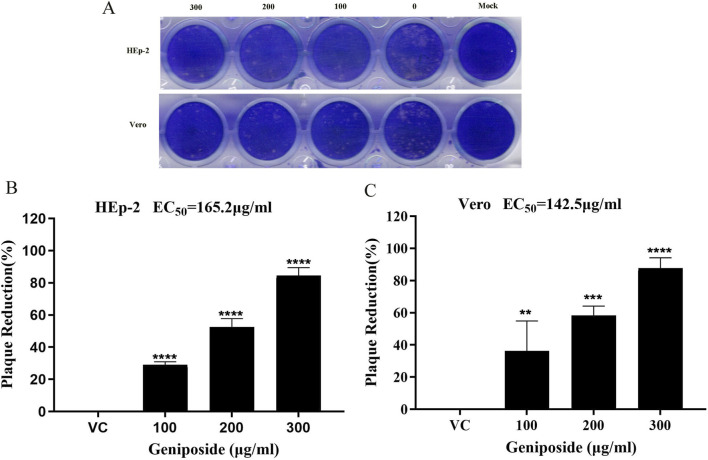
Geniposide suppresses the development of CVB3 viral plaques *in vitro* **(A)** Scanned visualization of CVB3 plaques in HEp-2 and Vero cells. **(B)** The plaque percentage relative to the virus control in HEp-2 cells. **(C)** The plaque percentage relative to the virus control in Vero cells. (n = 3; *p < 0.05, **p < 0.01, ***p < 0.001, ****p < 0.0001 vs. the virus control group).

Plaque reduction assay results (Figure 3A) showed that compared with the virus control group, the number of plaques in the geniposide-treated group significantly decreased with increasing drug concentration, showing a concentration-dependent inhibitory trend, indicating that geniposide can effectively inhibit plaque formation induced by Coxsackievirus infection. Analyzed by GraphPad Prism 5.0 software, the 50% effective concentrations (EC50) of geniposide against Coxsackievirus were 165.2 μg/mL (95% CI: 142.4–191.5 μg/mL) in HEp-2 cells and 142.5 μg/mL (95% CI: 108.5–187.2 μg/mL) in Vero cells (Figures 3B,C). The selectivity index (SI = CC50/EC50) of geniposide against CVB3 was calculated to be 3.57 (589.9/165.2) in HEp-2 cells and 3.67 (523.5/142.5) in Vero cells, indicating that geniposide possesses certain antiviral selectivity and low cytotoxicity *in vitro*. Collectively, these results confirm that geniposide exhibits a definite inhibitory effect against coxsackievirus *in vitro*, with comparable antiviral activity in the 2 cell models.

### 3.4 Results of network pharmacology analysis

**FIGURE 4:**
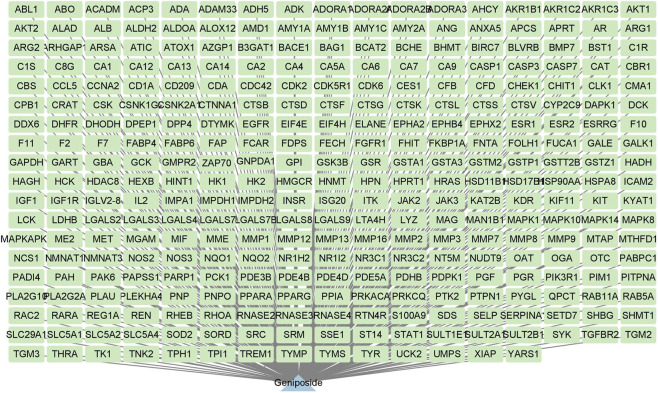
Action targets of the active component geniposide.

The active components and targets of geniposide were screened and obtained using network pharmacology, and a “component-target” network diagram was successfully constructed and analyzed (Figure 4). The results showed that a total of 320 drug targets of geniposide were identified.

### 3.5 Acquisition of CVB3-related targets and construction of PPI network for geniposide against CVB3

**FIGURE 5:**
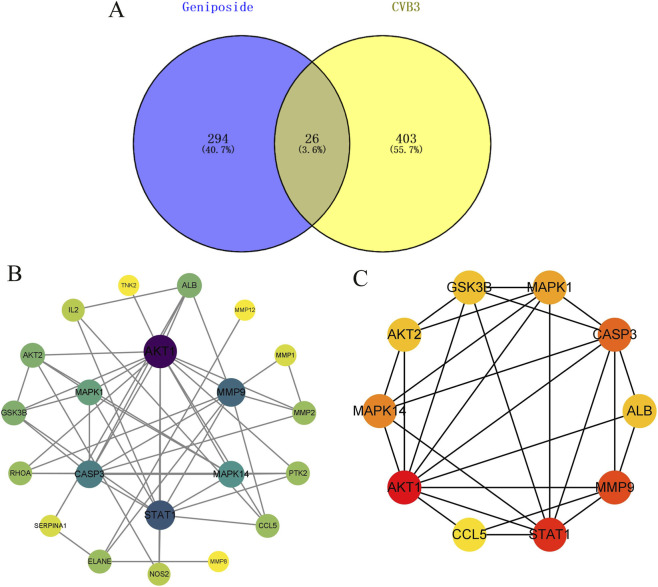
Venn diagram and PPI network for geniposide in treatment of CVB3. **(A)** Venn diagram of geniposide and CVB3-related targets. **(B)** PPI diagram of geniposide’s anti-CVB3 targets. **(C)** Top 10 core targets.

After sorting and deduplication in the GeneCards and OMIM databases, 429 CVB3-related disease targets were screened, and a Venn diagram of predicted drug and disease targets was generated (Figure 5A). Intersection targets were imported into the String database with the highest confidence threshold of 0.7 to obtain PPI network information, which was then imported into Cytoscape to generate a visual target map (Figure 5B). The top 10 core targets were screened, mainly including AKT1, STAT1, MMP9, CASP3, MAPK14, MAPK1, ALB, GSK3B, AKT2, and MMP2 (Figure 5C).

### 3.6 Enrichment analysis of intersection targets

**FIGURE 6:**
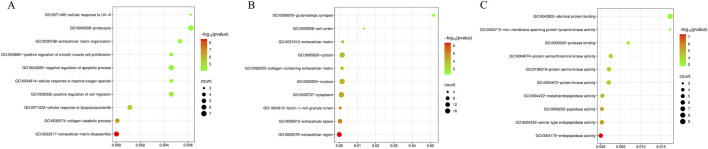
GO enrichment analysis of intersection targets. **(A)** GO BP Enrichment. **(B)** GO CC Enrichment. **(C)** GO MF Enrichment.

The 26 intersection targets obtained by the Venny tool were imported into the David database for GO gene function enrichment analysis, resulting in 114 entries related to biological processes (BP), 29 to cellular components (CC), and 55 to molecular functions (MF). Based on the GO analysis results, the top 10 entries in BP, CC, and MF were selected to draw enrichment maps (Figures 6A–C) to reveal the main functional mechanisms of geniposide against CVB3. In terms of biological processes, the anti-CVB3 effect of geniposide mainly involves extracellular matrix decomposition, collagen catabolic processes, cellular response to lipopolysaccharide, and positive regulation of cell migration. At the cellular component level, its effects are related to the extracellular region, extracellular space, ficolin-1-rich granule lumen, and cytoplasm. In terms of molecular functions, it is mainly associated with endopeptidase activity, serine-type endopeptidase activity, peptidase activity, and metalloendopeptidase activity.

### 3.7 KEGG pathway enrichment analysis

**FIGURE 7:**
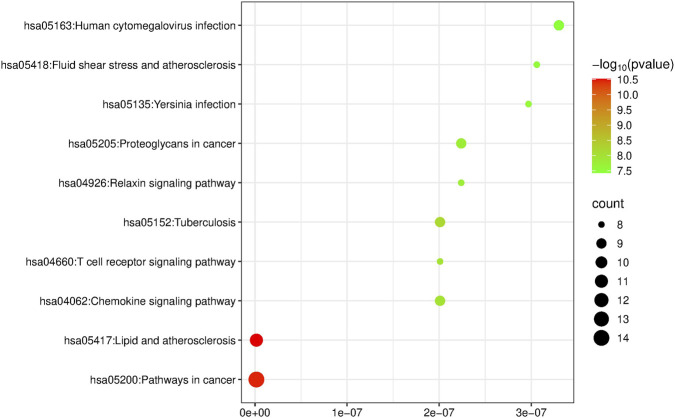
KEGG enrichment analysis of intersection targets.

KEGG pathway enrichment analysis identified 114 related signaling pathways, mainly involving lipid and atherosclerosis, pathways in cancer, tuberculosis, chemokine signaling pathway, T cell receptor signaling pathway, proteoglycans in cancer, relaxin signaling pathway, and *Yersinia* infection. Using P-values as the screening criterion, the top 10 pathways were selected to draw an enrichment map (Figure 7). The results indicate that geniposide may exert its anti-CVB3 effect by regulating these related signaling pathways.

### 3.8 Results of molecular docking

Six key core targets—AKT1, STAT1, MMP9, CASP3, MAPK14, and MAPK1—were selected for molecular docking verification with geniposide. The results showed that the binding energies of geniposide to these targets were −7.9, −7.8, −8.1, −7.3, −7.4, and −7.1 kcal/mol, respectively. Generally, a binding energy less than −5 kcal/mol indicates strong binding activity between the ligand and receptor. In this study, the binding energies of geniposide with the six core targets were all below −7 kcal/mol, predicting that geniposide could form stable binding conformations with these targets.

**FIGURE 8:**
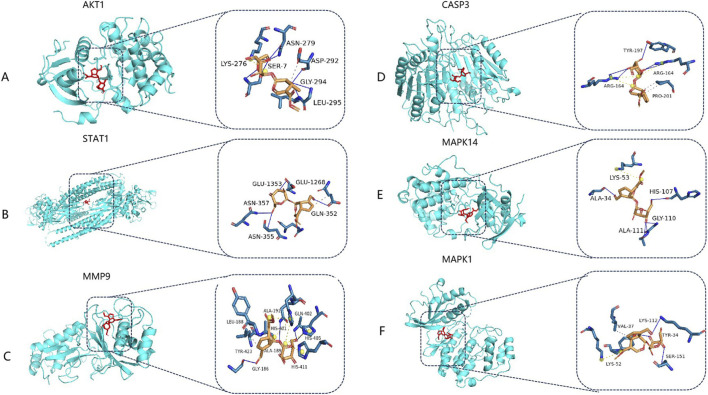
Binding pattern of geniposide to AKT1 protease **(A)**, STAT1 protease **(B)**, MMP9 protease **(C)**, CASP3 protease **(D)**, MAPK14 protease **(E)** and MAPK1 protease **(F)**. **(A)** Binding pattern of geniposide to AKT1. **(B)** Binding pattern of geniposide to STAT1. **(C)** Binding pattern of geniposide to MMP9. **(D)** Binding pattern of geniposide to CASP3. **(E)** Binding pattern of geniposide to MAPK14. **(F)** Binding pattern of geniposide to MAPK1.

This provides a theoretical molecular basis for the potential anti-CVB3 activity of geniposide and also supports the subsequent experimental verification of target interactions (Table 1; Figure 8).

**TABLE 1:** Molecular docking results of geniposide with core targets (kcal/mol).

Target	Binding energy
AKT1	−7.9
STAT1	−7.8
MMP9	−8.1
CASP3	−7.3
MAPK14	−7.4
MAPK1	−7.1

## 4 Discussion

Coxsackievirus B3 (CVB3) is a major pathogen causing viral myocarditis and dilated cardiomyopathy, with diseases induced by its infection posing significant harm to adolescents (Zhang et al., 2023). Currently, there are no specific therapeutic drugs or effective preventive vaccines clinically, so exploring new anti-CVB3 drugs has important theoretical and practical value. This study is the first to demonstrate that geniposide inhibits CVB3 replication *in vitro* in a concentration-dependent manner. Using network pharmacology combined with molecular docking, we predicted its potential targets and preliminary mechanism, which opens a new avenue for the development of anti-coxsackievirus drugs.

*In vitro* experimental results showed that geniposide alleviated the cytopathic effect in CVB3-infected HEp-2 and Vero cells in a concentration-dependent manner and significantly reduced viral plaque formation. The 50% effective concentrations (EC50) of geniposide against HEp-2 cells and Vero cells were 165.2 μg/mL and 142.5 μg/mL, respectively. Moreover, geniposide exhibited low cytotoxicity in both cell lines and a favorable selectivity index, indicating that geniposide possesses promising *in vitro* anti-CVB3 activity and acceptable safety.This finding further expands the antiviral spectrum of geniposide. Previous studies have confirmed that geniposide inhibits influenza virus and white spot syndrome virus (WSSV) (Huang et al., 2022; Zhang et al., 2024), whereas the present study demonstrates for the first time that geniposide exerts an inhibitory effect against CVB3, a member of the Enterovirus genus, providing new evidence for its application in the prevention and treatment of viral diseases. Compared with reported natural anti-CVB3 compounds, geniposide shows a moderate level of anti-CVB3 potency among natural products. Its potency is lower than those of dehydrosuccinic acid (6.77–11.57 μg/mL), berberine (37.18 μg/mL), and Selaginella moellendorffii extract (41 μg/mL), but superior to that of Scutellaria baicalensis extract, which only shows significant activity at 400 μg/mL (Yin et al., 2014; Dai et al., 2017; Fu et al., 2019; Li J. et al., 2025). Notably, geniposide features multi-target effects including antiviral, anti-inflammatory, and antioxidant activities, which are highly compatible with the pathological characteristics of CVB3-induced myocarditis, making it an ideal lead compound for structural modification.

Geniposide is a water-soluble iridoid glycoside compound, with an oral bioavailability of only 9.67% in rats, which represents a key bottleneck for its clinical translation. This constituent must be hydrolyzed by intestinal bacterial β-glucosidase into genipin before it can be absorbed, and it is rapidly eliminated from plasma, with nearly complete clearance within 12 h (Wang et al., 2014; Li et al., 2024; Zhang et al., 2025). Geniposide is widely distributed *in vivo*, with the highest accumulation in the kidneys, followed by the spleen, liver, heart, and other tissues, suggesting a potential risk of cumulative hepatotoxicity and nephrotoxicity at high doses. Its metabolism mainly depends on the intestinal flora and hepatic enzyme systems, and its metabolites are primarily excreted via the kidneys (Liu et al., 2022; Li et al., 2024).Therefore, the present study only verified the *in vitro* anti-CVB3 activity of geniposide. The main limitations are its relatively high *in vitro* EC50, which restricts its direct clinical translation as a single agent, and the lack of studies on *in vivo* antiviral efficacy, tissue targeting validation, and pharmacokinetic experiments. Future research may focus on structural modification of geniposide to screen highly active derivatives with low EC50; adopt nanodelivery systems and other technologies to improve its *in vivo* bioavailability and myocardial tissue targeting; perform *in vivo* experiments in CVB3-induced myocarditis animal models to evaluate its adjuvant therapeutic effect in combination with other low-toxicity compounds; and optimize the administration route of geniposide for clinical application.

Network pharmacology analysis further revealed the multi-target characteristics of geniposide against CVB3. By screening the intersection of drug and disease targets, core targets such as AKT1, STAT1, MMP9, CASP3, MAPK14 (p38 MAPK), and MAPK1 (ERK2) were found to be involved in its anti-CVB3 process. Molecular docking verified that geniposide forms stable bindings with these targets (binding energy < −7 kcal/mol), which not only supports the rationality of network pharmacology predictions but also suggests that these targets may serve as the potential molecular basis for the antiviral activity of geniposide. However, this study did not benchmark the docking scores against known ligands of the above targets, and the dynamic stability of geniposide-target binding conformations over time still requires further validation.

Further bioinformatics analysis of the core targets, combined with network pharmacology and molecular docking results, predicted that the anti-CVB3 mechanism of geniposide may involve the following pathways:

MAPK1/ERK2 and MAPK14/p38 MAPK are key members of the MAPK family, where ERK2 primarily regulates cell proliferation, differentiation, and survival, while p38 MAPK plays a critical role in mediating apoptosis and inflammatory responses (Ronkina and Gaestel, 2022). Studies have confirmed that CVB3 infection can activate ERK and p38 MAPK, promoting viral replication and inflammatory damage, while inhibiting the activity of these kinases can significantly reduce viral yield and alleviate myocardial lesions (Jensen et al., 2013; He et al., 2019). Studies by Li et al. have confirmed that dehydrotumulosic acid (DA) can exert antiviral activity against coxsackieviruses by activating the glucocorticoid receptor (GR) and thereby inhibiting the MAPK/ERK signaling pathway (Li J. et al., 2025). Berberine limits CVB3 replication by inhibiting JNK and p38 MAPK activation, all indirectly supporting that geniposide may act through the MAPK signaling pathway (Dai et al., 2017). Scutellaria baicalensis extract (SBE) exerts inhibitory effects on Coxsackievirus B3 (CVB3) replication by modulating the AKT and p38 signaling pathways, thereby effectively alleviating CVB3-induced myocarditis in both *in vitro* and *in vivo* models (Fu et al., 2019). In this study, the high-affinity binding of geniposide to MAPK1 and MAPK14 suggests that it may exert antiviral effects by potentially inhibiting the phosphorylation of ERK and p38 MAPK, thereby blocking signal-dependent steps in viral replication. These findings require further validation by time-dependent drug administration assays and phosphorylation level detection experiments.

PI3K/Akt signaling pathway: AKT1, a core molecule of the PI3K/Akt pathway, whose activation is crucial for efficient CVB3 replication (Esfandiarei et al., 2004). Studies have shown that CVB3 infection can induce Akt phosphorylation, thereby promoting viral protein synthesis and cell apoptosis by regulating downstream molecules such as mTOR and 4EBP1(Li et al., 2013). Moreover, the PI3K/Akt/mTOR signaling pathway is involved in autophagy induced by CVB3 infection (Chang et al., 2017). The stable binding between geniposide and AKT1 predicted that it may inhibit AKT1 activity, interfere with the metabolic demands of viral replication, and reduce apoptosis in infected cells. The precise timing and stages at which it regulates the viral life cycle remain to be further confirmed.

STAT1-mediated immune regulation: STAT1 is a key transcription factor in the type I interferon signaling pathway; its nuclear translocation can initiate the expression of antiviral genes (e.g., ISG) to inhibit viral replication. However, CVB3 infection can negatively regulate STAT1 through activating the STAT3-SOCS3 axis, impairing host immune responses (Liang et al., 2024). This study found that geniposide can target STAT1, suggesting that it may enhance STAT1 nuclear translocation and downstream antiviral gene expression, restoring the host’s innate immune defense function. This inference requires further verification by stage-specific experiments.

Regulation of apoptosis and inflammation: CASP3 (caspase 3) is a key executor of cell apoptosis (Asadi et al., 2022), while MMP9 (matrix metalloproteinase 9) is involved in tissue remodeling during inflammatory responses (Regalado and Balogh, 2024). CVB3 infection can induce myocardial cell apoptosis by activating CASP3(Wu et al., 2020) and upregulate MMP9 to exacerbate inflammatory damage (Yang et al., 2011). The binding of geniposide to these two targets may directly inhibit the apoptotic program and the release of inflammatory mediators, thereby reducing virus-induced cell damage, which may provide a new perspective for its application in the treatment of viral myocarditis. Notably, the enrichment of MMP9 (matrix metalloproteinase 9) may be related to CVB3-induced degradation of myocardial extracellular matrix, and geniposide’s inhibition of MMP9 activity may reduce virus-induced myocardial damage, providing a new perspective for its application in viral myocarditis treatment.

This study represents a preliminary *in vitro* exploratory investigation on the anti-CVB3 activity of geniposide, and several limitations should be acknowledged as follows: First, this study only evaluated the antiviral activity of geniposide using phenotypic assays, including MTT cytotoxicity testing, observation of cytopathic effect, and plaque reduction assay. It did not include direct quantitative approaches to monitor viral replication, such as qRT-PCR and Western blot for CVB3 RNA and VP1 protein expression, and thus could not fully delineate the intracellular replication kinetics of CVB3. Second, Network pharmacology and molecular docking only provided *in silico* predictions of core targets and binding potential, whereas experimental evidence supporting target regulation and pathway modulation (including MAPK/AKT phosphorylation, STAT1 nuclear translocation, and CASP3 activation) was lacking in this study. This study also did not clarify the time window at which geniposide exerts its anti-CVB3 effect, nor its specific regulatory roles in key stages of the CVB3 life cycle (entry, replication, assembly, and release). All mechanistic inferences above require verification by refined experimental studies. The molecular docking results only reflected static binding capacity at the simulation level, without benchmarking against known ligands or validation of dynamic binding stability via molecular dynamics simulation. Third, the HEp-2 and Vero cells used in this study are highly susceptible to CVB3 infection, exhibit stable infection phenotypes, and consistently produce typical cytopathic effects (CPE) and plaque formation. Thus, they represent a reliable *in vitro* platform for the preliminary screening of anti-CVB3 agents. However, both HEp-2 and Vero cell lines are defective in interferon-mediated antiviral responses, and cannot reflect the actual function of the STAT1-mediated interferon signaling pathway within the *in vivo* immune microenvironment during geniposide against CVB3.Therefore, this study only demonstrates that geniposide exhibits significant concentration-dependent anti-CVB3 activity *in vitro* in HEp-2 and Vero cells. The *in vitro* mechanism of action only provides a hypothetical framework for subsequent *in vivo* studies, and cannot directly extrapolate its *in vivo* therapeutic efficacy, effective dosage, or actual clinical value against CVB3-induced myocarditis in humans. Fourth, this study lacked a well-validated broad-spectrum antiviral drug as a positive control, making it difficult to assess the relative potency of geniposide against CVB3.

Based on the limitations mentioned above, the follow-up research plan of this study is proposed as follows. To begin with, the levels of CVB3 nucleic acid and VP1 protein will be detected by qRT-PCR and Western blot, so as to quantitatively analyze the inhibitory effect of geniposide on viral replication kinetics. Subsequently, Western blot, immunofluorescence staining and pathway inhibitor rescue assays will be applied to verify the regulatory effects of geniposide on core targets and signaling pathways, including phosphorylated MAPK1, phosphorylated MAPK14, phosphorylated AKT1, cleaved CASP3, and STAT1 nuclear translocation. Detailed time-course studies and viral life-cycle stage validation will be performed to determine the optimal time window and stage specificity of geniposide against CVB3, further refining the mechanistic evidence of its antiviral activity. Parallel molecular docking will be carried out between geniposide and classic known ligands or positive inhibitors of core targets to establish a benchmark comparison of binding affinities. In addition, 50- to 100-nanosecond molecular dynamics simulations will be conducted to analyze the dynamic stability of geniposide-target interactions and validate the reliability of the binding conformations. Next, the anti-CVB3 activity of geniposide will be evaluated in immunocompetent cell models, such as primary cardiomyocytes and bone marrow-derived macrophages, as well as in in vivo animal models using mice with CVB3-induced viral myocarditis. The role of the STAT1-mediated interferon antiviral pathway, together with the *in vivo* efficacy and safety of geniposide, will be validated. Meanwhile, a positive control drug, such as ribavirin, will be included to compare plaque inhibition efficiency, cytoprotective effects and viral replication inhibition. Molecular docking and network pharmacology analysis of ribavirin on core anti-CVB3 targets will also be performed to assess the relative potency of geniposide. Moreover, structure-activity relationship studies of geniposide will be conducted to optimize its pharmacological activity and lower its effective working concentration. Finally, further investigation will be carried out on the antiviral specificity of geniposide against other coxsackievirus serotypes and non-enteroviruses.

Studies have shown that low-dose geniposide is associated with high safety and no obvious toxic reactions (Shan et al., 2017). The present study confirmed that geniposide exhibited favorable *in vitro* antiviral activity against CVB3 with low cytotoxicity. The half cytotoxic concentration (CC50) of geniposide in HEp-2 cells and Vero cells was much higher than its half effective concentration (EC50) against CVB3. However, with further research and exploration of potential clinical applications, the potential side effects of geniposide cannot be ignored. Studies have indicated that high-dose or long-term administration of geniposide may induce hepatotoxicity in animal models, and excessive geniposide may also cause mild metabolic burden in hepatocytes of rodents. This phenomenon may be related to the metabolic conversion of geniposide into genipin in the liver (Shan et al., 2017; Li et al., 2019). Therefore, this study only evaluated the cytotoxicity of geniposide in two immortalized cell lines under short-term culture conditions, lacking systematic safety evaluation data such as *in vivo* acute/chronic toxicity, organ histopathology, and drug-drug interactions. In addition, the antiviral effect of geniposide was only verified in HEp-2 and Vero cell lines; its therapeutic efficacy and safety in animal models of CVB3-induced myocarditis remain to be further confirmed. In the follow-up preclinical research, we will utilize CVB3-infected murine and rat models to carry out multi-aspect safety evaluations of geniposide. The aim will be to identify its safe dosage range, target organ toxicity, and reversible toxic features, which will furnish robust preclinical evidence for advancing geniposide as a potential anti-CVB3 drug candidate.

## 5 Conclusion

This study demonstrates for the first time that geniposide exhibits *in vitro* anti-CVB3 activity in both HEp-2 and Vero cells. Geniposide dose-dependently alleviated CVB3-induced cytopathic effects, inhibited viral plaque formation, and showed low cytotoxicity with favorable antiviral selectivity. Using network pharmacology and molecular docking, we predicted six core potential targets of geniposide against CVB3: AKT1, STAT1, MMP9, CASP3, MAPK14 and MAPK1. Geniposide displayed stable binding potential with these targets, providing a clear hypothetical framework for subsequent mechanistic validation. Further studies can verify the regulatory roles of the above pathways using techniques such as Western blotting and gene knockout. *In vivo* experiments should be performed to optimize the formulation and administration route of geniposide, promoting its translational application in the adjuvant treatment of CVB3-induced viral myocarditis. These findings support further preclinical investigation of geniposide as a potential lead compound against CVB3 and provide novel targets and directions for the development of anti-coxsackievirus drugs.

## Data Availability

The original contributions presented in the study are included in the article/supplementary material, further inquiries can be directed to the corresponding author.
